# Monitoring disease progression in childhood bronchiectasis

**DOI:** 10.3389/fped.2022.1010016

**Published:** 2022-09-16

**Authors:** Kathryn A. Ramsey, André Schultz

**Affiliations:** ^1^Wal-yan Respiratory Research Centre, Telethon Kids Institute, University of Western Australia, Perth, WA, Australia; ^2^Respiratory Medicine, Perth Children's Hospital, Perth, WA, Australia

**Keywords:** bronchiectasis, children, imaging, lung function, exacerbations, symptoms, biomarkers, monitoring

## Abstract

Bronchiectasis (not related to cystic fibrosis) is a chronic lung disease caused by a range of etiologies but characterized by abnormal airway dilatation, recurrent respiratory symptoms, impaired quality of life and reduced life expectancy. Patients typically experience episodes of chronic wet cough and recurrent pulmonary exacerbations requiring hospitalization. Early diagnosis and management of childhood bronchiectasis are essential to prevent respiratory decline, optimize quality of life, minimize pulmonary exacerbations, and potentially reverse bronchial disease. Disease monitoring potentially allows for (1) the early detection of acute exacerbations, facilitating timely intervention, (2) tracking the rate of disease progression for prognostic purposes, and (3) quantifying the response to therapies. This narrative review article will discuss methods for monitoring disease progression in children with bronchiectasis, including lung imaging, respiratory function, patient-reported outcomes, respiratory exacerbations, sputum biomarkers, and nutritional outcomes.

## Introduction

Bronchiectasis is by nature a progressive disease thought be driven by a vicious cycle of impaired mucociliary clearance, inflammation, and infection on the surface of the lower airways. The rate of progression depends on the underlying cause as well as various factors related to the patient and their external environment. Acute exacerbations of bronchiectasis represent sudden worsening of the disease, often with permanent detrimental consequences and hastening of disease progression.

Improving disease monitoring was recently identified as a major theme when clinical and research priorities for bronchiectasis in children and young people were mapped (1). Disease monitoring potentially allows for (1) the early detection of acute exacerbations, facilitating timely intervention, (2) tracking the rate of disease progression for prognostic purposes, and (3) quantifying the response to therapies. A disease that primarily involves the lower airways, bronchiectasis causes respiratory symptoms and affects lung structure and function. However, bronchiectasis also has systemic effects, influencing quality of life and daily functioning. This review will discuss the monitoring of disease progression in childhood bronchiectasis, starting with symptoms before covering methods to measure lung structure, function, infection, inflammation, patient-reported outcomes and exacerbations. This narrative review will focus on non-cystic fibrosis bronchiectasis in general and does not elaborate on specific underlying diseases that might cause bronchiectasis.

## Cough and sputum

Inflammation and infection of the airway surface cause secretions, which typically present as wet-sounding cough. A wet cough in a patient who usually does not have a wet cough is suggestive of a respiratory exacerbation. Children who have a daily wet cough when clinically stable tend to have more advanced disease; in these individuals, an increase in cough or sputum production represents a flare-up of disease. In children old enough to expectorate, the increased secretions that cause wet cough can be evaluated through the measurement of sputum characteristics. Sputum volume or weight is typically measured after a particular intervention (e.g., airway clearance therapy) or over a time period (typically 24 h) (2, 3). A sputum purulence score has also been developed and used in clinical trials (4, 5). Sputum volume in adults with exacerbations of bronchiectasis reduces after treatment with intravenous antibiotics (3). The frequency of cough can be measured objectively with electronic cough monitors but such measurement is not always practical. Diary cards completed by children with the assistance of their parents have been shown to correlate well with cough frequency measured electronically (6). Whilst symptoms are important and relatively easy to measure, other measures such as lung imaging provide more objective and accurate measurements of structural disease.

## Lung imaging

Imaging is used to measure the extent of structural lung disease in bronchiectasis. Chest x-ray is not sensitive enough to detect and monitor bronchiectasis in children. High-resolution computed tomography (HRCT) of the chest is the gold standard method to confirm bronchiectasis. The current European Respiratory Society guidelines for the management of children and adolescents with bronchiectasis recommend that multidetector chest CT scans with HRCT be used instead of conventional HRCT alone for diagnosing bronchiectasis when available (7), based on data reporting greater sensitivity in adult patients (8, 9). The guidelines also recommend that a broncho-arterial ratio (BAR) > 0.8 be used to define abnormality instead of the adult cut-off (>1–1.5), based on evidence that BAR correlates with age and is significantly lower in children and adolescents compared with adults in healthy individuals (10).

In addition to being critical to the diagnosis of bronchiectasis, chest CT offers an objective and sensitive method to assess and monitor disease severity in children. Studies have shown that bronchiectasis and associated structural abnormalities on CT can remain static, show improvement, or even completely resolve over time in response to early effective treatment in childhood (11, 12). Chest CT is more sensitive than lung function to monitor disease in children, with bronchiectasis detected on CT in the presence of normal spirometry (13). Quantitative scoring methods specific for pediatric bronchiectasis are needed to monitor small changes in disease extent over time. Longitudinal imaging studies are required to understand the natural progression of bronchiectasis development and progression, to identify potential precursors of bronchiectasis such as bronchial wall thickening, mucus plugging, and trapped gas (a marker of small airway disease), and to inform clinical guidelines as to the appropriate intervals between HRCT scans for clinical disease surveillance.

Improvements in CT technology and imaging algorithms now allow HRCT at radiation doses that are substantially lower than only a couple of decades ago (14), but disease monitoring with no radiation is still preferable. Lung magnetic resonance imaging (MRI) has the potential for a radiation-free method to assess disease progression over time. Traditionally, the use of MRI in the context of pediatric bronchiectasis has been limited by low spatial resolution when imaging the lungs and the need for a patient to remain still for extended periods during image capture. Advances in lung MRI using ultra-short echo time sequences allow for reduced motion artifact, increased parenchymal signal, and improved spatial resolution (15). In addition, functional MRI scans can now be performed without the need for contrast agents or breathing maneuvers and allow for visualization and quantification of ventilation and perfusion defects in the lung (16). These techniques are sensitive to structural lung abnormalities and bronchiectasis in children and adolescents with cystic fibrosis (CF) and primary ciliary dyskinesia (17–19). Future work is needed to determine the sensitivity of lung MRI to detect bronchiectasis compared to HRCT and its clinical utility in monitoring disease progression in childhood with bronchiectasis.

It is important to note that in some settings and countries, the accessibility, cost, and availability of gold standard lung imaging and general anesthesia for children are limited. This impedes the radiological diagnosis of bronchiectasis and assessment of disease extent needed to guide clinical management. Chest imaging allows for accurate measurement of structural lung disease but does not measure lung function. Novel technologies that might allow the measurement of both lung structure and function are in development but are not ready for clinical use yet (20, 21). In the meantime, lung function measurements, discussed next, are still core to the monitoring of bronchiectasis.

## Lung function

The preservation of lung function is a primary objective in managing children and adolescents with bronchiectasis (7). Spirometry is the most widely used method for measuring lung function in bronchiectasis. Low spirometry outcomes in childhood and early adulthood are associated with significant respiratory and cardiovascular morbidity and mortality in later life (22). Adults with bronchiectasis who developed a chronic cough in childhood had significantly lower FEV_1_ than those with adult-onset symptoms, suggesting that unmanaged bronchiectasis in childhood will result in progressive lung function decline (23).

The typical lung function trajectory in children with bronchiectasis has not yet been accurately characterized. Longitudinal studies with serial lung function measurements in childhood have shown conflicting results. One study from New Zealand reported that children with bronchiectasis had significant airway obstruction that deteriorates over time (24). Other studies from the UK and Australia have shown that spirometry outcomes are relatively stable or significantly improve with specialist treatment over a period of 3–5 years (25–28). These differences are likely attributable to age, radiological disease severity, ethnicity, and management strategies. However, all of these studies have been retrospective with relatively small sample sizes. One prospective, long-term, multicentre study of First Nations children with chronic suppurative lung disease (CSLD) or bronchiectasis from Australia, Alaska, and New Zealand receiving specialized care showed that the majority of children had stable lung function within the normal range (29). However, children with bronchiectasis had lower lung function than those with CSLD, and many children reported ongoing respiratory symptoms and school absences, indicating ongoing underlying disease (29). Additional prospective studies are needed to understand the biological factors driving lung function trajectories in childhood and adolescence.

Studies in adults have shown that frequency of pulmonary exacerbations, colonization with *Pseudomonas aeruginosa*, and systemic inflammation are associated with lung function decline (30). Pediatric cohort studies show that frequent hospitalized exacerbations, later diagnosis of bronchiectasis, and later referral to specialist care are associated with lung function decline in childhood (27, 28). Poorer spirometry outcomes are more likely in children with bronchiectasis in developing countries (31), First Nations children (32), and those with poor growth and nutrition (27, 28). However, there are no clear associations between lung function outcomes and underlying cause, sex, or radiological disease severity. These data suggest that early diagnosis and optimal management of bronchiectasis in childhood can attenuate lung function deterioration into early adulthood.

While spirometry is the standard method for assessment of lung function in the clinical setting, alternative methods may be more sensitive or provide complementary information when monitoring early lung disease in children with bronchiectasis. Spirometry outcomes can be within the normal range in children with both CF and non-CF bronchiectasis, despite extensive structural lung damage on high-resolution chest CT (13). Chest CT scans stabilize and improve in children with bronchiectasis following intensive treatment (11). However, as serial chest CT scans are not routinely performed in the surveillance of non-CF bronchiectasis, it is unclear whether improvements in spirometry outcomes correspond to reduced radiological disease severity.

The lung clearance index (LCI) from the multiple breath washout technique has potential as a sensitive clinical endpoint to monitor disease progression in childhood bronchiectasis. In children with CF, LCI declines earlier than FEV_1_, correlates with pulmonary inflammation and the extent of lung disease on chest CT, and is more responsive to treatment than FEV_1_ (33–36). Studies in adults with bronchiectasis have shown that LCI is a repeatable and sensitive marker of structural lung disease on chest CT (37, 38). However, it is not clear whether LCI is responsive to short-term interventions or sensitive to mild lung disease in childhood bronchiectasis (39, 40). Further work is needed to identify lung function tests that can monitor disease progression and predict disease outcomes in pediatric bronchiectasis (1).

## Infection

Over time, children with bronchiectasis are increasingly likely to become chronically infected with pathogenic bacteria. Chronic infection with *Pseudomonas aeruginosa* is associated with an increased frequency of exacerbations, lower lung function, and more severe bronchiectasis (41). A reduced density of *P. aeruginosa* (brought about by inhaled tobramycin) in the sputum of adults with bronchiectasis who are chronically infected is associated with reduced symptoms and exacerbations (42). Similarly, inhaled ciprofloxacin reduces the density of *Pseudomonas* in sputum and also reduces exacerbations in adults with bronchiectasis (43, 44). In a randomized controlled trial in children with bronchiectasis and chronic infection, 12 weeks of nebulised gentamycin resulted in reduced bacterial growth (of *H. influenzae*) and bacterial density, although lung function remained unchanged (45). Therefore, the presence and density of pathogens in sputum are related to bronchiectasis disease and response to treatment i.e., antibiotics. In clinical practice, the effect of antibiotics should always be weighed up against the potential emergence of new pathogens and antimicrobial resistance (44, 46).

## Inflammatory biomarkers

Inflammation is fundamental to the pathophysiology of bronchiectasis. Airway inflammation is best measured through bio sampling of the lower airways, which can be challenging in young children. Young children are typically unable to expectorate sputum, and older children with mild disease might not expectorate unless they are experiencing exacerbations. Studies in young children with CF suggest that oropharyngeal swabs and induced sputum do not represent the lower airways in young children (47–49), whilst the invasive nature and cost related to bronchoalveolar lavage limits its routine use for disease monitoring. Hence, our knowledge of the role of inflammation in children with bronchiectasis is based mostly on small, cross-sectional studies, retrospective chart reviews, or extrapolation of data from studies in adults. Sputum induction methods are likely to benefit this area of research in the future as it has in children with CF (50), although the role of sputum induction in young children who are unable to expectorate is debatable (49).

Neutrophilic inflammation and associated proinflammatory cytokines (e.g., IL-8, TNFa, IL1-B, IL-6) and proteases (e.g., neutrophil elastase) have been found in children with bronchiectasis (51–54). In adults, levels of neutrophilic inflammation are elevated in both stable disease and during pulmonary exacerbations (55–57). Inflammatory markers are associated with poor lung function, the extent of bronchiectasis, respiratory symptoms, and airway mucus concentration (51, 58–60). Sputum neutrophil elastase is an important biomarker of disease severity and can predict the risk of exacerbations in adult bronchiectasis (61). In addition to neutrophilia, a small proportion of children with bronchiectasis show evidence of eosinophilic inflammation (53, 54). In adults with bronchiectasis, eosinophilia is associated with more severe disease (62). Eosinophilic inflammation in this context likely represents a complex inflammatory pathway associated with multiple etiologies including virus and parasite infections, coexistent asthma, and hypersensitivity to fungi.

Bronchiectasis is associated with both localized inflammation in the lungs and with systemic inflammation. Systemic inflammation in adults with bronchiectasis is reflected by elevated inflammatory cells (total white cells, neutrophils) and serum mediators (e.g., TGF-B, C-reactive protein) and correlates with lung function decline (30). A small study in children with bronchiectasis has shown elevated systemic proinflammatory lymphocytes (TNF-a, IFN-y, perforin, and granzyme) (54), however, more studies are needed to identify sensitive and non-invasive markers of low-level inflammation (both airway and systemic) in children.

## Patient-reported outcomes

Patient-reported outcomes measure a patient's perception of their health and capture facets of disease not measured through conventional methods. The most common patient-reported outcome measure is health related quality of life. At least 12 different health-related quality of life questionnaires have been developed for use in adults with bronchiectasis (63). The development and validation of such tools take time and are labor intensive. A parent-proxy quality of life questionnaire for pediatric chronic cough has been developed, validated (64, 65) and used in clinical trials of children with bronchiectasis (66). An 8-point version of the questionnaire has also been successfully used in First Nation contexts (67). Patient-reported outcomes augment objective measures such as lung function and markers of inflammation to provide a more holistic measure of patient health.

## Respiratory exacerbations

Exacerbations of bronchiectasis are important events associated with reduced quality of life, reduced school attendance, and drops in lung function that could be permanent. Therefore, the timely and accurate diagnosis of exacerbations is an essential part of clinical care. Parents have identified improved management of exacerbations as one of the top three priorities (1).

Exacerbations in children are characterized by an increase in symptoms and signs. Wet cough and cough severity are the best predictors of exacerbations (68). Most exacerbations present with wet cough and increased sputum production, or worsening of cough, while more severe presentations can also present with a spectrum of fatigue, dyspnoea, crepitations, wheeze, chest pain, haemoptysis and fever. A European Respiratory Society Task Force recently recommended that, for clinical purposes, an exacerbation should be defined as “an increase in respiratory symptoms (predominantly increased cough +/- increased sputum quantity and/or purulence) for >3-days,” and a severe exacerbation diagnosed when there is dyspnoea or hypoxia, irrespective of symptom duration (7). These definitions differ from those used in adults because children with bronchiectasis are different from their adult counterparts in terms of their physiology and immunological responses (69), microbiota in the lower airways (52), and severity of bronchiectasis. The European Respiratory Society Task Force also wisely pointed out that changes in auscultation and radiological findings should not be relied on when diagnosing exacerbations, and children with neurodevelopmental disorders might present with subtle and individualized symptoms (7).

Exacerbations become more frequent as disease progresses and are arguably the most important outcome to measure in clinical trials of children with bronchiectasis. Successful clinical trials in children have used pragmatic definitions for mild exacerbations such as the respiratory episodes treated with antibiotics (70), or more refined definitions like “an increase in cough frequency, a change in character of the cough from dry to wet, or an increase in sputum volume or purulence for at least 3 days” (66, 71). Severe exacerbations usually include hospitalization in the definition. Drop in spirometry values is a less sensitive measure of exacerbation, and hence clinical trials tend to focus more on the duration of symptoms or hospitalization.

## Discussion

Bronchiectasis in children can be monitored through a range of modalities. Each modality has unique strengths and weaknesses, and some very promising modalities like LCI require further validation before use in clinical practice can become routine. The monitoring of bronchiectasis in children is complicated by a lack of non-invasive measures of infection and inflammation. Research to develop such measures is sorely needed.

Bronchiectasis is a condition greatly in need of therapies that will slow down disease progression, reduce exacerbations, and improve the quality of life of patients. Clinical trials will be critically important in developing such therapies. The choice of outcome measures is fundamental to clinical trial design. The Children's Bronchiectasis Education Advocacy and Research Network (Child-BEAR-Net) is currently developing a set of core outcome measures to this purpose (72).

## Author contributions

Both authors listed have made a substantial, direct, and intellectual contribution to the work and approved it for publication.

## Funding

AS received salary and research support from a MRFF Investigator Grant (APP1193796) and an unrestricted grant from Mineral Resources Ltd.

## Conflict of interest

The authors declare that the research was conducted in the absence of any commercial or financial relationships that could be construed as a potential conflict of interest.

## Publisher's note

All claims expressed in this article are solely those of the authors and do not necessarily represent those of their affiliated organizations, or those of the publisher, the editors and the reviewers. Any product that may be evaluated in this article, or claim that may be made by its manufacturer, is not guaranteed or endorsed by the publisher.
